# Mastic Oil Inhibits the Metastatic Phenotype of Mouse Lung Adenocarcinoma Cells

**DOI:** 10.3390/cancers3010789

**Published:** 2011-02-23

**Authors:** Heleni Loutrari, Sophia Magkouta, Andreas Papapetropoulos, Charis Roussos

**Affiliations:** 1 “G.P. Livanos and M. Simou Laboratories”, Evangelismos Hospital, Department of Critical Care and Pulmonary Services, School of Medicine, University of Athens, 3 Ploutarchou Street, 10675 Athens, Greece; E-Mails: smagkouta@med.uoa.gr (S.M.); croussos@med.uoa.gr (C.R.); 2 Laboratory of Molecular Pharmacology, Department of Pharmacy, University of Patras, 26504 Patras, Greece; E-Mail: apapapet@upatras.gr

**Keywords:** lung cancer, prevention, mastic plant essential oil, invasion, motility, adhesion, angiogenesis

## Abstract

Mastic oil from *Pistacia lentiscus* variation *chia*, a natural combination of bioactive terpenes, has been shown to exert anti-tumor growth effects against a broad spectrum of cancers including mouse Lewis lung adenocarcinomas (LLC). However, no studies have addressed its anti-metastatic actions. In this study, we showed that treatment of LLC cells with mastic oil within a range of non-toxic concentrations (0.01–0.04% v/v): (a) abrogated their Matrigel invasion and migration capabilities in transwell assays; (b) reduced the levels of secreted MMP-2; (c) restricted phorbol ester-induced actin remodeling and (d) limited the length of neo-vessel networks in tumor microenvironment in the model of chick embryo chorioallantoic membrane. Moreover, exposure of LLC and endothelial cells to mastic oil impaired their adhesive interactions in a co-culture assay and reduced the expression of key adhesion molecules by endothelial cells upon their stimulation with tumor necrosis factor-alpha. Overall, this study provides novel evidence supporting a multipotent role for mastic oil in prevention of crucial processes related to cancer metastasis.

## 1. Introduction

Lung cancers are among the most mortal cancer types characterized by rapid metastasis and frequent resistance to current chemotherapy regimens and radiotherapy [1]. Therefore, novel agents preventing both cancer onset and metastatic spreading are urgently required for their treatment. Recently much attention has been focused on phytochemicals, *i.e.*, bioactive compounds isolated from plants, due to their low toxicity and multiple chemopreventive/chemotherapeutic actions that have been attributed to the fine tuning of intracellular and intercellular signal transduction pathways regulating cell homeostasis [2,3]. Current data support that natural combinations of phytochemicals often possess enhanced reactivity compared to isolated substances due to their additive and/or synergistic interactions [4]. Mastic gum from *Pistacia lentiscus* var. *chia* and its essential oil, two natural products traditionally used for their food flavoring and medicinal properties, seem to be promising in this respect as they contain a wide spectrum of bioactive components, mainly terpenes [5-7] and have proved to be safe in humans and experimental animals at oral daily doses as high as 40–60 mg mastic gum/kg of body weight for a period of 3–6 weeks [8-11]. Among mastic oil components, the isoprenoid perillyl alcohol (POH), an established inhibitor of the mevalonate biosynthetic pathway [12], has proved to efficiently attenuate tumor growth [13,14], metastasis [15] and angiogenesis [16] and it is undergoing phaseI/II clinical trials [17,18]. In concert, mastic gum and mastic oil have been also shown to exert *in vitro* anti-tumor growth activities against various cancer types including human prostate, leukemia and colon cancers [19-22] within a range of effective non-toxic concentrations (0.001–0.6% v/v). Using a Lewis lung adenocarcinoma (LLC) transplantation model we have further demonstrated that mastic oil (45 mg/kg body weight, i.p., every other day for about three weeks) can significantly limit tumor expansion in syngeneic mice without toxicity, by targeting *in vivo* cancer cell apoptosis, tumor-associated neovascularization and inflammation, in part through negative modulation of Ras/RhoA GTPases and NF-kappaB-dependent gene transcription [23]. Recently, by combining high-throughput transcriptomic technology and bioinformatics on mastic oil-treated LLC cells we have been able to identify a number of target genes, such as those encoding PTEN, E2F7, HMOX1 (up-regulation) and NOD1 (down-regulation) and provide insights into the pathways involved in oncogenic growth inhibition [24]. Although some of the reported actions could also support a potential role of mastic oil in metastasis prevention, this issue has not yet been investigated.

Metastasis unequivocally marks an advanced and generally incurable stage of tumor progression by which cells from the primary tumor invade the basement membrane, enter the circulation through newly formed blood vessels and disseminate to distant sites [25,26]. In the present work we explored—for the first time—the consequences of mastic oil treatment on this complex cascade using highly metastatic LLC cells in a series of assays simulating stages that critically influence cell invasiveness and tumor interactions with neighboring vasculature. POH was included for comparison as a reference bioactive component.

## 2. Results and Discussion

Despite the substantial advancement in cancer treatment, metastasis remains the main cause of cancer fatality [27]. Discovering new relatively non-toxic agents capable of preventing this lethal condition is an important challenge with major implications for clinical practice. In this study, by combining a number of biological assays, we were able to demonstrate for the first time that mastic oil, a dietary plant extract with established anti-tumor growth activity, can negatively control pivotal steps of metastasis.

### 2.1. Viability of Mastic Oil-Treated LLC Cells

Using different experimental tumor growth models, we have previously shown that mastic oil combines pleiotropic anti-cancer actions with low toxicity even at relatively high concentrations [20,23]. In this study, we initially wished to confirm cell viability in LLC cultures under treatment conditions (cell density, culture media, concentrations of test agents, incubation time) simulating those of applied experimental protocols. Using an MTT-based method, we found that compared to vehicle control, mastic oil at concentrations ranging from 0.01 to 0.04 % v/v and POH at 0.5–1.0 mM for various time periods up to 48 h, did not significantly altere (p > 0.05) the number of living cells (Table 1). These concentrations were used in all the different assays. Viability tests were also performed in endothelial cells (EC) and no toxicity was observed (data not shown).

### 2.2. Mastic Oil Inhibits Tumor Cell Invasiveness

**Figure 1.**
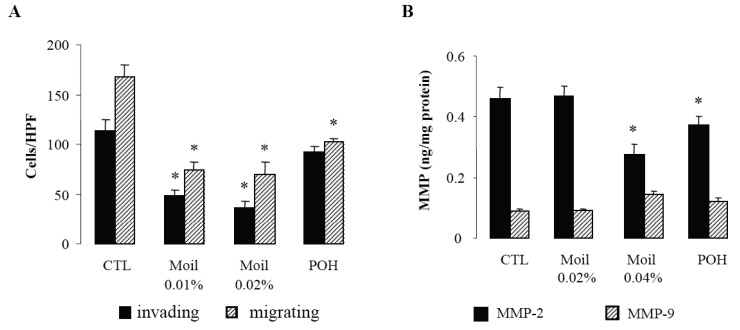
Mastic oil effects on tumor cell invasion/migration and MMP expression. (**A**) Mastic oil attenuates tumor cell invasion and migration. Serum starved LLC cells were treated with Moil (0.01–0.02% v/v), POH (0.5mM) or vehicle (CTL) for 2 h and then loaded onto BD Matrigel Invasion or Control chambers. The lower chambers were filled with complete medium containing test agents and plates were incubated for 20 h. Migrating cells were counted (200 X magnification, 10 HPF/membrane) and results are expressed as mean ± SEM; n = 9, *P < 0.05 from vehicle. (**B**) Mastic oil effects on MMP-2 and MMP-9 expression. Serum starved LLC cultures were treated with Moil (0.02–0.04 % v/v), POH (1 mM) or vehicle (CTL) for 48 h and supernatants were analyzed for MMP-2 and MMP-9 levels by ELISA. Results were normalized to total protein and are expressed as mean ± SEM; n = 12, *P < 0.05 from vehicle.

Since invasion of the extracellular matrix (ECM) by cancer cells is essential for their dissemination, we first examined in a transwell *in vitro* assay the capability of mastic oil-treated tumor cells to invade and move through Matrigel-coated filters. Migration of tumor cells through identical uncoated membranes was also assessed in parallel using similar experimental conditions. As shown in Figure 1A, mastic oil even at low concentrations (0.01% and 0.02% v/v) significantly limited tumor cell invasiveness and migration capabilities indicating that it could target both the enzymatic machinery involved in ECM degradation and the cell motility mechanism [28]. Notably, POH at 0.5 mM (Figure 1A) and 1.0 mM (not shown) although affecting LLC cell migration, was not able to cause any significant change in the number of invading LLC cells, thus underlining the contribution of additional bioactive ingredients into mastic oil.

In view of the pivotal role of type IV collagenases, matrix metalloproteinases 2 (MMP-2) and 9 (MMP-9) on ECM degradation and local invasion by solid cancers [29-31], we next evaluated the effect of mastic oil on their basal expression by LLC cells. In agreement with previous reports [29] we were able to detect only low constitutive levels of these enzymes (Figure 1B). Nevertheless, treatment with mastic oil further reduced the expression of MMP-2 but only when applied at the highest concentration (0.04% v/v). Similarly, POH decreased MMP-2 levels only at 1 mM (0.5 mM did not cause any significant effect, data not shown). Although these results do not directly correlate MMP-2 reduction with the observed inhibition of *in vitro* anti-invasive activity (Figure 1A), which could be achieved at lower concentrations of mastic oil (0.01–0.02% v/v), they are, however, indicative of a negative modulation of signaling pathways controlling MMP-2 expression. This finding may have therapeutic relevance as MMP-2, unlike many other MMPs, is constitutively expressed by a wide range of malignant cell types and its over-expression has been suggested to be of independent prognostic value in cancer patients [31]. As for MMP-9, its secreted levels were not significantly affected by mastic oil nor POH. However, the chance of detecting some decrease was technically limited by the fact that the levels of MMP-9 at basal state of LLC cells were quite near the detection threshold of the applied assay.

### 2.3. Mastic Oil Reduces Actin Remodeling

**Figure 2.**
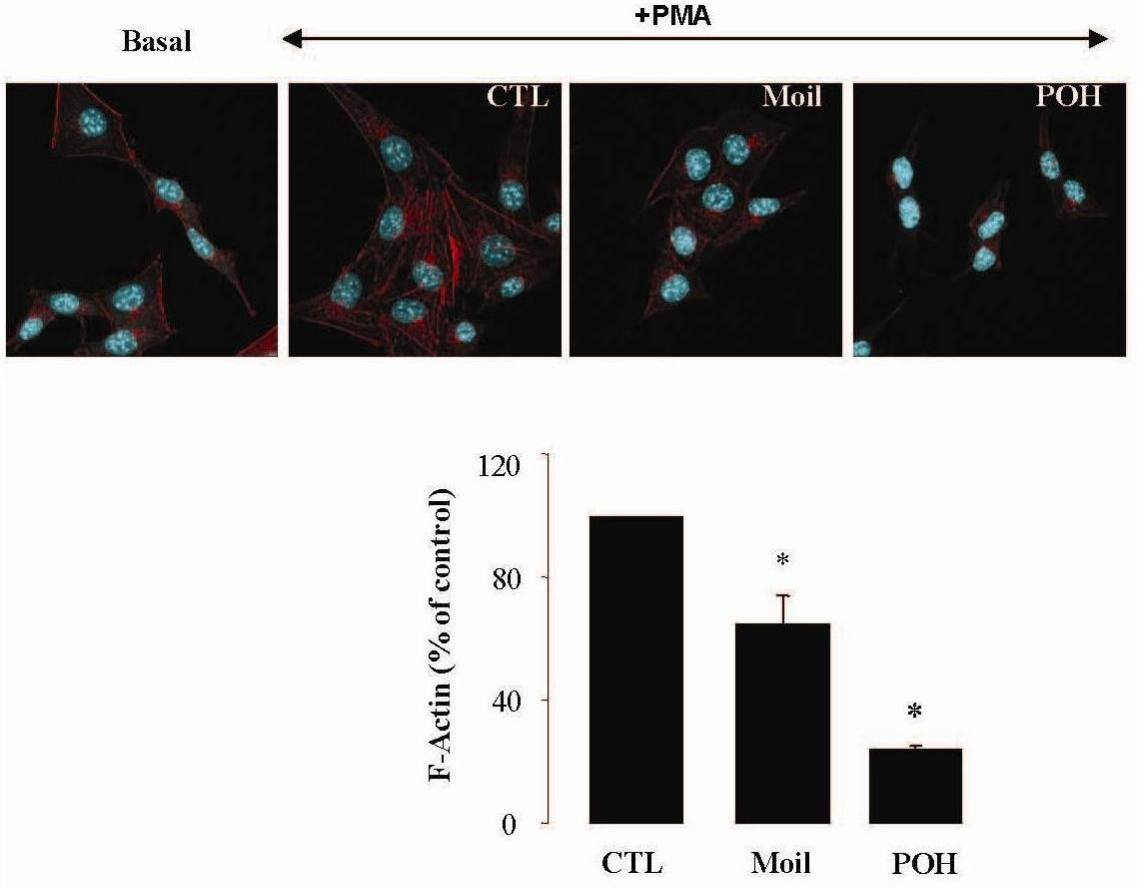
Mastic oil inhibits actin polymerization in PMA-induced LLC cells. Serum-starved LLC cells were treated with Moil (0.04% v/v), POH (1.0 mM) or vehicle (CTL) for 3 h and then induced by 1 μM PMA or vehicle (basal) for 30 min. Cells were fixed and F-actin was visualized by phalloidin staining. Top panel: representative micro-photographs (630 X magnification); bottom panel: graphs from image analysis of F-actin staining normalized to cell numbers and expressed as mean (% of control) ± SEM; n = 96–160, *P < 0.05.

It has been well established that the process of cell motility requires the dynamic organization of the actin cytoskeleton, which in turn involves the polymerization and depolymerization of actin filaments in response to chemotactic stimuli [32]. Therefore, to explore the basis of mastic oil-mediated inhibitory action on LLC cell motility, we subsequently investigated its potential effects on actin cytoskeleton remodeling using phorbol 12-myristate 13-acetate (PMA) as inducer, a tumor promoter signaling phospholipid [33]. Actin rearrangement in mastic oil (0.01–0.04 % v/v), POH (0.5–1.0 mM) or vehicle treated LLC cells was assessed before and after addition of PMA by means of immunofluorescent labeling and confocal microscopy of filamentous (F)-actin. As shown in Figure 2 (which illustrates the effects in the high concentration range), although non-induced LLC cells displayed low basal levels of polymerized F-actin, exposure to PMA caused a prominent staining especially at the periphery, indicating an increase in *de novo* actin polymerization. Importantly, pre-incubation with mastic oil and POH resulted in a significant attenuation of F-actin fiber formation in PMA-stimulated cells thus suggesting the ability of these agents to attenuate pathways triggering changes in the actin cytoskeleton such as those induced by the used phospholipid [32-34].

### 2.4. Mastic Oil Inhibits Tumor Interactions with Vascular Microenvironment

**Figure 3.**
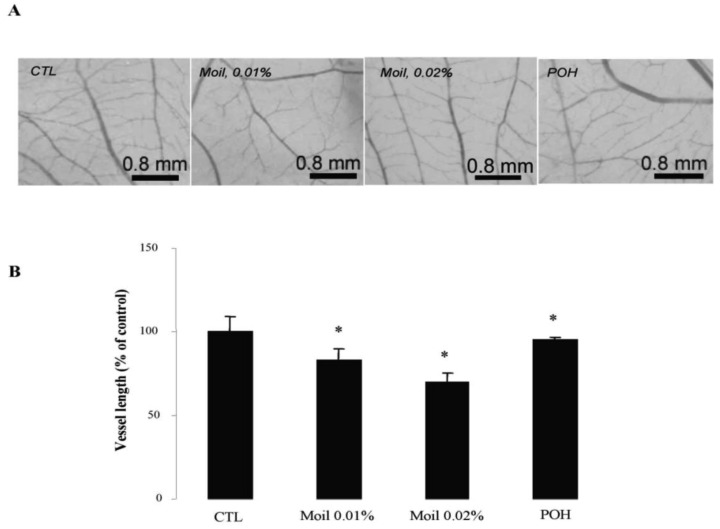
Mastic oil attenuates tumor-related angiogenesis. Tumor cells pre-treated with vehicle (CTL), Moil (0.01-0.02 % v/v) or POH (0.5 mM) for 2 h were applied onto the CAM and further incubated for 48 h at 37 °C in the presence of test agents. (**A**) Representative photographs of vessel networks in the CAM; (**B**) Graphs from image analysis of vessel network length (mean % of control ± SEM, n = 30, *P<0.05 from vehicle.

We subsequently wished to address the consequences of mastic oil treatment on critical connections between tumor cells and the neighboring vasculature leading to tumor-related angiogenesis, endothelial adhesion and vascular penetration [25,35]. Previous experimental evidence from our laboratory indicated an inhibitory role of mastic oil on vascularization of LLC tumors and on the expression of two important chemotactic/pro-angiogenic mediators, namely vascular endothelial growth factor and monocyte chemoattractant protein-1 [23]. Herein, by using an alternative tumor growth model in chicken embryo chorioallantoic membrane (CAM), which recapitulates several of the *in vivo* tumor-host interactions [16,36], we were able to directly estimate the length of the vascular network in tumor microenvironment. As revealed by image analysis of pictures taken two days after the initial inoculation of tumor cells on the CAM, mastic oil treatment at 0.01% to 0.04% v/v reduced the length of the microvessel networks surrounding LLC implants in a concentration-dependent manner, compared to the vehicle control, whereas POH (0.5 and 1.0 mM) had a similar but weaker effect. Figure 3 shows the effects of test agents at the lower concentration range. Although MMP-2 has been shown to promote tumor-associated vascular remodeling [30,31], the anti-angiogenic effects presented in Figure 3 may not be related to the decrease of secreted MMP-2 by mastic oil as it was mediated only at the highest test concentration (0.04% v/v, Figure 1B).

**Figure 4.**
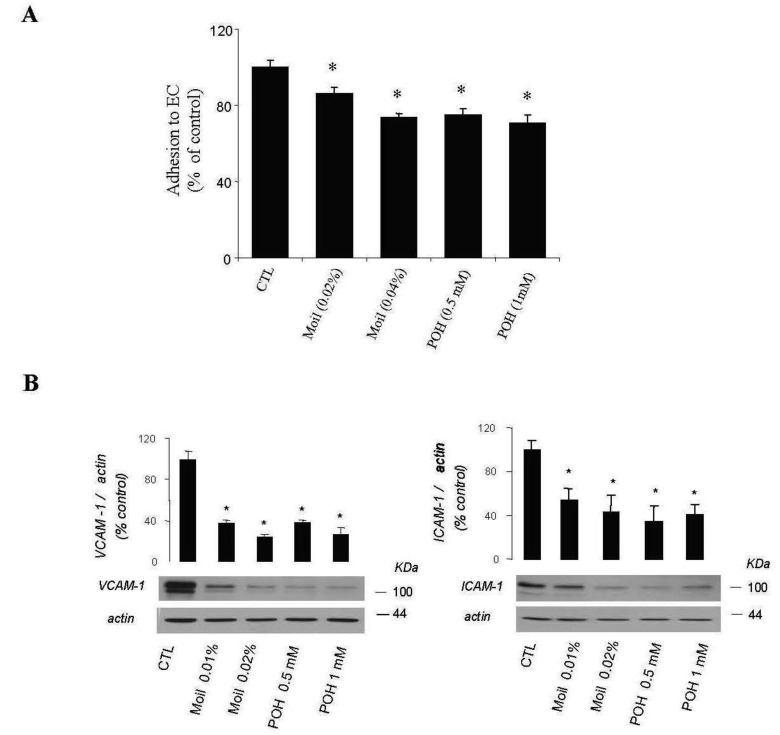
(**A**) Mastic oil inhibits LLC cell adhesion to endothelial cell (EC) monolayers. Fluorescently labeled tumor cells treated with Moil (0.02–0.04% v/v), POH (0.5–1 mM) or vehicle (CTL) for 2 h were loaded onto confluent EC monolayers treated similarly and co-cultures were further incubated in the presence of test agents for 1 h. After washing, LLC adherent cells were lysed and fluorescence was measured at 485–535 nm. Results are expressed as means ± SEM; n = 18. (**B**) Mastic oil decreases the expression of endothelial ICAM-1 and VCAM-1. HUVEC monolayers were treated with Moil (0.01–0.02% v/v), POH (0.5–1 mM) or vehicle (CTL) for 2 h and then TNF-α (10 ng/mL) was added and incubated for 4 h. Cell lysates were analyzed for the presence of ICAM-1 and VCAM-1 by Western blotting. Blots are representative of three independent experiments. Total densitometry data were normalized to β-actin and expressed as mean % of control ±SEM, *P < 0.05.

Furthermore, by using a simple quantitative assay, we examined whether mastic oil was able to modify tumor cell adhesiveness to EC monolayers. Results were similar for EC originated from two different vascular beds (human umbilical vein and bovine coronary vein, HUVEC and CVEC, respectively) and are presented in total. As shown in Figure 4A, pre-treatment of LLC and EC with mastic oil at the high concentration range (0.02–0.04% v/v) reduced the number of tumor cells adhering to endothelial monolayers in a concentration-dependent manner. In line with this data, Western blot analysis of mastic oil-treated EC revealed a dose-dependent reduction, at an overlapping concentration range (0.01-0.02% v/v), in the expression of two important adhesion mediators, ICAM-1 and VCAM-1 [37], upon induction with an inflammatory cytokine known to potentate *in vivo* cancer metastasis, tumor necrosis factor-α (TNF-α, Figure 4B) [38]. POH (0.5-1 mM) displayed a similar activity pattern to mastic oil in both assays (Figure 4A and 4B).

Taken together, our data indicate that mastic oil could limit the metastatic potential of LLC cells through negative regulation of MMP-2 expression, actin cytoskeleton remodeling and tumor endothelial adhesion. Although additional experiments are required to provide further mechanistic insights, several of these actions may be associated with the established inhibitory effects of mastic oil on small RhoA GTPase [20,23] and NF-*κ*B [22-24] signaling known to control relevant metastasis-promoting processes [39,40]. Furthermore, up-regulation of tumor suppressor *pten* in LLC cells treated by mastic oil, previously revealed by genomic microarray analysis [24], may also provide an additional mechanistic link, as PTEN over-expression has been shown to suppress the process of lung cancer invasion [41]

## 3. Experimental Section

### 3.1. Phytochemicals

Mastic oil was from Chios Gum Mastic Growers Association (Chios, Greece) and POH was from Fluka (Buchs, Switzerland). Chemical composition of mastic oil was in accordance with published data [5,6]. Different batches of extract displayed significant reproducibility in all types of assays. Selection of bioactive concentrations was based on previous studies [16,20,23] and verified by preliminary experiments. POH concentration (0.5–1 mM) corresponded always to molar excess compared to its content (about 1%) into the examined concentrations of mastic oil. Working dilutions contained up to 0.1% DMSO (Sigma, St. Louis, MO, USA).

### 3.2. Cell Culture and Viability Assay

LLC cells (American Type Culture Collection, Manassas, VA, USA) were cultured according to [23]. Human umbilical vein and bovine coronary vein endothelial cells (HUVEC and CVEC, respectively) were isolated and maintained as described before [16,42]. Cell viability was monitored under experimental conditions specified for each biological test by the methylthiazoletetrazolium (MTT) assay (Sigma, St. Louis, MO, USA) and by trypan blue exclusion. Briefly for MTT assay, 90% confluent LLC cultures grown in 96-well plates were serum-starved for 24 h. Test agents were then added (mastic oil at 0.01–0.04 % v/v, POH at 0.5-1.0 mM or DMSO vehicle) and incubation continued for various times up to 48 h. After the exposure period, MTT (5 mg/mL) was added and cells were further incubated for 4 h at 37 °C. The MTT formazan crystals were solubilised by the addition of 0.1N HCl in anhydrous isopropanol and the absorbance was measured on a microtiter plate reader at 595 nm with correction at 630 nm. Sample absorbance was correlated with cell number using a reference standard curve.

### 3.3. Invasion and Migration Assays

Invasion and migration of LLC cells were assayed using 8μm-pore size membrane BD BioCoat Matrigel Invasion and Control Chambers respectively (BD Biosciences, Erembodegen, Belgium). Confluent tumor cell cultures grown in 6-well plates were serum-starved for 24 h and then treated with mastic oil (0.01–0.04% v/v), POH (0.5–1.0 mM), or DMSO for 2 h. Cells were then trypsinized and 0.5 mL of suspension containing 3 × 10^4^ cells were loaded on the upper compartment, the lower chamber was filled with DMEM containing 10% FCS (or serum-free medium in controls) in the presence of test agents or vehicle and the plate was incubated for 20 h at 37 °C. Membranes were then fixed and stained using the Rapi- Diff II kit (Bios Europe, Lancashire, UK). Cells located in randomly chosen fields on the lower surface of the membrane were counted in blind on a microscope (Å = 200; 10 high power field/membrane).

### 3.4. Determination of MMP Expression

LLC cells were seeded onto 12-well plates at a density of 1 × 10^5^ cells/well and 24 h later the cells were treated with mastic oil (0.01–0.04% v/v), POH (0.5–1.0 mM) or DMSO-vehicle for 24–48 h in serum-free medium. Culture supernatants were analyzed for the presence of total MMP-2 and MMP-9 by ELISA (R&D Systems, Minneapolis, MN, USA) according to the manufacturers' instructions. Results were normalized to total protein.

### 3.5. Immunofluorescent Detection of F-Actin

Localization of F-actin was performed by immunofluorescent cell labeling as described before [43] Briefly, tumor cells (1 ×10^5^ cells/mL) were plated on 1% gelatin-coated glass culture coverslips in complete medium for 24 h. The medium was replaced with serum-free DMEM containing 0.25% BSA and cultures were further incubated for 24 hours. Cells were then treated with mastic oil (0.01–0.04 %v/v), POH (0.5–1.0 mM) or DMSO for 3 h and then 1 μM PMA (Sigma, St. Louis, MO, USA) or vehicle was added for 30 min. Cells were then washed twice with PBS, fixed with 3.7% formaldehyde solution for 5 min, lysed with 1% Triton X-100 and dyed with 5 units Alexafluor 488 phalloidin (Invitrogen, Carlsbad, CA, USA). Coverslips were mounted onto slides with Prolong Gold antifade reagent with DAPI (Invitrogen, Carlsbad, CA, USA) for nuclear staining. Pictures from non-overlapping random fields were taken under a confocal laser scanning microscope (DMI400B, Leica Microsystems AG, Wetzlar, Germany) using a 630× oil immersion objective. For quantification of F-actin, photographs were analyzed with ImageJ software (NIH Image, Bethesda, MD, USA) and data normalized to cell number.

### 3.6. Tumor Angiogenesis Model in the Chorioallantoic Membrane (CAM)

To asses neo-vessel formation in tumor microenvironment, we used the CAM of the chick embryo model in a modified version that combined two previously described protocols [16,36]. Briefly, 5 × 10^5^ tumor cells that had been pre-treated for 2 h with mastic oil (0.01–0.04% v/v, POH (0.5–1 mM) or vehicle (0.05% DMSO), were applied on the exposed upper part of the membrane on day 9 of embryo development, onto an area of 1 cm^2^ restricted by a plastic ring. After incubation of eggs for 48 hours at 37 °C in the presence of test compounds, the upper CAM was fixed in situ, excised from the eggs and pictures were taken from the tumor periphery through a stereoscope equipped with a digital camera. The total vessel length was measured using image analysis software (Scion Image, Scion Corporation, Frederick, MD) according to [16].

### 3.7. Tumor-Endothelial Cell Adhesion Assay

Adhesion capability of LLC cells to EC monolayers was evaluated as described previously [44] with some modifications. Tumor cells pre-treated with mastic oil (0.01–0.04% v/v), POH (0.5–1 mM), or DMSO vehicle for 3 h at 37 °C, were labeled with 10 μg/mL of 2,7-bis-(2-carboxyethyl)-5-(6)-carboxyfluorescein acetoxymethyl ester (BCECF-AM; Sigma, St. Louis, MO) for 30 min at 37 °C, washed twice with PBS and collected. Subsequently, 1 × 10^5^ labeled cells were seeded onto EC grown in 96-well plates similarly pre-treated with test agents and co-cultures incubated for 1 h. After three washes with PBS, cells were solubilized by 0.5% Triton X-100 and fluorescence of lysates measured at 485/535 nm excitation/emission wavelengths. Numbers of adherent cells were evaluated using a reference standard curve.

### 3.8. Detection of ICAM-1 and VCAM-1 by Western Blotting

EC were treated with medium containing various concentrations of mastic oil (0.01–0.04% v/v), POH (0.5–1.0 mM) or DMSO vehicle (0.1%) for 2 h. Subsequently, tumor necrosis factor-alpha (TNF-α, 10 ng/mL) or PBS was added and cultures were further incubated for 4 h. Cells were then solubilized and lysates were resolved by 10% SDS-PAGE as described previously [20] using anti-human ICAM-1, VCAM-1 (R&D Systems, Minneapolis, MN, USA) or anti-beta-actin (Chemicon, Temecula, CA, USA) antibodies. Gel-Pro Analyzer software (Media Cybernetics, Silver Spring, MD, USA) was used for densitometry analysis of blots and results were normalized to beta-actin.

### 3.9. Data Analysis and Statistics

Data are presented as means ± SEM of the indicated number of observations. Statistical comparisons between groups were performed using one-way ANOVA followed by a *post hoc* test as appropriate. Differences among means were considered significant when P < 0.05.

## 4. Conclusions

Overall, in this study, by using experimental models and assays evaluating critical processes of the metastatic cascade, we provided novel evidence for the inhibitory actions and some potential underlying mechanisms of mastic oil against cancer cell metastatic spreading.

## Figures and Tables

**Table 1.** Viability of Lewis lung adenocarcinoma (LLC) cells after treatment.

**Incubation Time (h)**	**Mastic Oil Concentration (% v/v)**			**POH Concentration (mM)**	
	**0.01**	**0.02**	**0.04**	**0.5**	**1.0**
6	99.6 ± 1.5	94.7 ± 1.6	95.8 ± 2.4	98.4 ± 2.0	94.7 ± 1.4
24	101.7 ± 1.7	98.2 ± 2.9	96.1 ± 2.5	100.7 ± 1.7	91.2 ± 1.0
48	102.6 ± 2.0	97.1 ± 2.4	93.3 ± 1.9	97.2 ± 2.5	92.1 ± 1.5

Confluent cultures of LLC cells grown in 96-well plates were serum-starved for 24 h and then treated with test agents or vehicle for 6-48 h. Results are expressed as mean % of control ± SEM, n = 12.

## References

[1] Jemal A., Siegel R., Ward E., Hao Y., Xu J., Murray T., Thun M.J. (2008). Cancer statistics, 2008. CA Cancer J. Clin..

[2] D'Incalci M., Steward W.P., Gescher A.J. (2005). Use of cancer chemopreventive phytochemicals as antineoplastic agents. Lancet Oncol..

[3] Surh Y.J. (2003). Cancer chemoprevention with dietary phytochemicals. Nat. Rev. Cancer.

[4] de Kok T.M., van Breda S.G., Manson M.M. (2008). Mechanisms of combined action of different chemopreventive dietary compounds: A review. Eur. J. Nutr..

[5] Papageorgiou V., Sagredos A., Moser R. (1981). GLC-MS computer analysis of the essential oil of mastic gum. Chim. Chronica. New Ser..

[6] Magiatis P., Melliou E., Skaltsounis A.L., Chinou I.B., Mitaku S. (1999). Chemical composition and antimicrobial activity of the essential oils of *Pistacia lentiscus* var. chia. Planta Med..

[7] Koutsoudaki C., Krsek M., Rodger A. (2005). Chemical composition and antibacterial activity of the essential oil and the gum of *Pistacia lentiscus* Var. chia. J Agric Food Chem.

[8] Bebb J.R., Bailey-Flitter N., Ala'Aldeen D., Atherton J.C. (2003). Mastic gum has no effect on Helicobacter pylori load in vivo. J. Antimicrob. Chemother..

[9] Kang J.S., Wanibuchi H., Salim E.I., Kinoshita A., Fukushima S. (2007). Evaluation of the toxicity of mastic gum with 13 weeks dietary administration to F344 rats. Food Chem. Toxicol..

[10] Kaliora A.C., Stathopoulou M.G., Triantafillidis J.K., Dedoussis G.V., Andrikopoulos N.K. (2007). Chios mastic treatment of patients with active Crohn's disease. World J. Gastroenterol..

[11] Doi K., Wei M., Kitano M., Uematsu N., Inoue M., Wanibuchi H. (2009). Enhancement of preneoplastic lesion yield by Chios Mastic Gum in a rat liver medium-term carcinogenesis bioassay. Toxicol. Appl. Pharmacol..

[12] Mo H., Elson C.E. (2004). Studies of the isoprenoid-mediated inhibition of mevalonate synthesis applied to cancer chemotherapy and chemoprevention. Exp. Biol. Med. (Maywood).

[13] Belanger J.T. (1998). Perillyl alcohol: Applications in oncology. Altern. Med. Rev..

[14] Crowell P.L. (1999). Prevention and therapy of cancer by dietary monoterpenes. J. Nutr..

[15] Teruszkin Balassiano I., Alves de Paulo S., Henriques Silva N., Curie Cabral M., Gibaldi D., Bozza M., da Fonseca CO., Da Gloria da Costa Carvalho M. (2002). Effects of perillyl alcohol in glial C6 cell line in vitro and anti-metastatic activity in chorioallantoic membrane model. Int. J. Mol. Med..

[16] Loutrari H., Hatziapostolou M., Skouridou V., Papadimitriou E., Roussos C., Kolisis F.N., Papapetropoulos A. (2004). Perillyl alcohol is an angiogenesis inhibitor. J. Pharmacol. Exp. Ther..

[17] da Fonseca C.O., Schwartsmann G., Fischer J., Nagel J., Futuro D., Quirico-Santos T., Gattass C.R. (2008). Preliminary results from a phase I/II study of perillyl alcohol intranasal administration in adults with recurrent malignant gliomas. Surg. Neurol..

[18] Matos J.M., Schmidt C.M., Thomas H.J., Cummings O.W., Wiebke E.A., Madura J.A., Patrick L.J., Crowell P.L. (2008). A pilot study of perillyl alcohol in pancreatic cancer. J. Surg. Res..

[19] He M.L., Yuan H.Q., Jiang A.L., Gong A.Y., Chen W.W., Zhang P.J., Young C.Y., Zhang J.Y. (2006). Gum mastic inhibits the expression and function of the androgen receptor in prostate cancer cells. Cancer.

[20] Loutrari H., Magkouta S., Pyriochou A., Koika V., Kolisis F.N., Papapetropoulos A., Roussos C. (2006). Mastic oil from Pistacia lentiscus var. chia inhibits growth and survival of human K562 leukemia cells and attenuates angiogenesis. Nutr. Cancer.

[21] Balan K.V., Prince J., Han Z., Dimas K., Cladaras M., Wyche J.H., Sitaras N.M., Pantazis P. (2007). Antiproliferative activity and induction of apoptosis in human colon cancer cells treated in vitro with constituents of a product derived from *Pistacia lentiscus* L. var. chia. Phytomedicine.

[22] He M.L., Li A., Xu C.S., Wang S.L., Zhang M.J., Gu H., Yang Y.Q., Tao H.H. (2007). Mechanisms of antiprostate cancer by gum mastic: NF-kappaB signal as target. Acta Pharmacol Sin.

[23] Magkouta S., Stathopoulos G.T., Psallidas I., Papapetropoulos A., Kolisis F.N., Roussos C., Loutrari H. (2009). Protective effects of mastic oil from Pistacia lentiscus variation chia against experimental growth of Lewis Lung Carcinoma. Nutr. Cancer.

[24] Moulos P., Papadodima O., Chatziioannou A., Loutrari H., Roussos C., Kolisis F.N. (2009). A transcriptomic computational analysis of mastic oil-treated Lewis lung carcinomas reveals molecular mechanisms targeting tumor cell growth and survival. BMC Med. Genomics.

[25] Folkman J. (2002). Role of angiogenesis in tumor growth and metastasis. Semin. Oncol..

[26] Steeg P.S. (2006). Tumor metastasis: mechanistic insights and clinical challenges. Nat. Med..

[27] Psaila B., Lyden D. (2009). The metastatic niche: Adapting the foreign soil. Nat. Rev. Cancer.

[28] Geho D.H., Bandle R.W., Clair T., Liotta L.A. (2005). Physiological mechanisms of tumor-cell invasion and migration. Physiology (Bethesda).

[29] Nabeshima K., Inoue T., Shimao Y., Sameshima T. (2002). Matrix metalloproteinases in tumor invasion: Role for cell migration. Pathol Int..

[30] Chakrabarti S., Patel K.D. (2005). Matrix metalloproteinase-2 (MMP-2) and MMP-9 in pulmonary pathology. Exp. Lung Res..

[31] Turpeenniemi-Hujanen T. (2005). Gelatinases (MMP-2 and -9) and their natural inhibitors as prognostic indicators in solid cancers. Biochimie.

[32] Olson M.F., Sahai E. (2009). The actin cytoskeleton in cancer cell motility. Clin. Exp. Metastasis.

[33] Downey G.P., Chan C.K., Lea P., Takai A., Grinstein S. (1992). Phorbol ester-induced actin assembly in neutrophils: Role of protein kinase C. J. Cell Biol..

[34] Nomura N., Nomura M., Sugiyama K., Hamada J. (2007). Phorbol 12-myristate 13-acetate (PMA)-induced migration of glioblastoma cells is mediated via p38MAPK/Hsp27 pathway. Biochem. Pharmacol..

[35] Wels J., Kaplan R.N., Rafii S., Lyden D. (2008). Migratory neighbors and distant invaders: Tumor-associated niche cells. Genes Dev..

[36] Zijlstra A., Mellor R., Panzarella G., Aimes R.T., Hooper J.D., Marchenko N.D., Quigley J.P. (2002). A quantitative analysis of rate-limiting steps in the metastatic cascade using human-specific real-time polymerase chain reaction. Cancer Res..

[37] Kobayashi H., Boelte K.C., Lin P.C. (2007). Endothelial cell adhesion molecules and cancer progression. Curr. Med. Chem..

[38] Balkwill F. (2009). Tumour necrosis factor and cancer. Nat. Rev. Cancer.

[39] Aggarwal B.B. (2004). Nuclear factor-kappaB: The enemy within. Cancer Cell.

[40] Aznar S., Fernandez-Valeron P., Espina C., Lacal J.C. (2004). Rho GTPases: Potential candidates for anticancer therapy. Cancer Lett..

[41] Hong T.M., Yang P.C., Peck K., Chen J.J., Yang S.C., Chen Y.C., Wu C.W. (2000). Profiling the downstream genes of tumor suppressor PTEN in lung cancer cells by complementary DNA microarray. Am. J. Respir. Cell Mol. Biol..

[42] Schelling M.E., Meininger C.J., Hawker J.R., Granger H.J. (1988). Venular endothelial cells from bovine heart. Am. J. Physiol..

[43] Patel T.R., Corbett S.A. (2004). Simvastatin suppresses LPS-induced Akt phosphorylation in the human monocyte cell line THP-1. J. Surg. Res..

[44] Vaporciyan A.A., Jones M.L., Ward P.A. (1993). Rapid analysis of leukocyte-endothelial adhesion. J. Immunol. Methods.

